# Effects of shepherds and dogs on livestock depredation by leopards (*Panthera pardus*) in north-eastern Iran

**DOI:** 10.7717/peerj.3049

**Published:** 2017-02-23

**Authors:** Igor Khorozyan, Mahmood Soofi, Mobin Soufi, Amirhossein Khaleghi Hamidi, Arash Ghoddousi, Matthias Waltert

**Affiliations:** 1 Workgroup on Endangered Species, J.F. Blumenbach Institute of Zoology and Anthropology, Georg-August-Universität Göttingen, Göttingen, Germany; 2 Department of Environmental Sciences, Gorgan University of Agricultural Sciences and Natural Resources, Gorgan, Iran; 3 Persian Wildlife Heritage Foundation, Tehran, Iran; 4 Conservation Biogeography Lab, Geography Department, Humboldt-Universität zu Berlin, Berlin, Germany

**Keywords:** Human-wildlife conflict, Carnivore conservation, Husbandry, Mitigation

## Abstract

Human-carnivore conflicts over livestock depredation are increasingly common, yet little is understood about the role of husbandry in conflict mitigation. As shepherds and guarding dogs are most commonly used to curb carnivore attacks on grazing livestock, evaluation and improvement of these practices becomes an important task. We addressed this issue by studying individual leopard (*Panthera pardus*) attacks on sheep and goats in 34 villages near Golestan National Park, Iran. We obtained and analyzed data on 39 attacks, which included a total loss of 31 sheep and 36 goats in 17 villages. We applied non-parametric testing, Poisson Generalized Linear Modelling (GLM) and model selection to assess how numbers of sheep and goats killed per attack are associated with the presence and absence of shepherds and dogs during attacks, depredation in previous years, villages, seasons, ethnic groups, numbers of sheep and goats kept in villages, and distances from villages to the nearest protected areas. We found that 95.5% of losses were inflicted in forests when sheep and goats were accompanied by shepherds (92.5% of losses) and dogs (77.6%). Leopards tended to kill more sheep and goats per attack (surplus killing) when dogs were absent in villages distant from protected areas, but still inflicted most losses when dogs were present, mainly in villages near protected areas. No other variables affected numbers of sheep and goats killed per attack. These results indicate that local husbandry practices are ineffectual and the mere presence of shepherds and guarding dogs is not enough to secure protection. Shepherds witnessed leopard attacks, but could not deter them while dogs did not exhibit guarding behavior and were sometimes killed by leopards. In an attempt to make practical, low-cost and socially acceptable improvements in local husbandry, we suggest that dogs are raised to create a strong social bond with livestock, shepherds use only best available dogs, small flocks are aggregated into larger ones and available shepherds herd these larger flocks together. Use of deterrents and avoidance of areas close to Golestan and in central, core areas of neighboring protected areas is also essential to keep losses down.

## Introduction

Conflicts between rural communities and mammalian carnivores are widespread, arising from depredation losses and threats to humans (Inskip & Zimmermann, 2009; Loveridge et al., 2010). These conflicts challenge biodiversity conservation because they often occur inside or near protected areas and involve threatened carnivore species (Miller, 2015). Although inflicted losses rarely exceed 2–3% of living stock, they can be economically substantial for individual households and thus stimulate retaliatory killing of carnivores, particularly big cats (Holmern, Nyahongo & Røskaft, 2007; Dar et al., 2009; Bauer, De Iongh & Sogbohossou, 2010; Tumenta et al., 2013). Persecution by humans drives big cats towards extinction as five out of seven species of these carnivores are classified by the IUCN Red List of Threatened Species as Vulnerable to Endangered (tiger *Panthera tigris*, lion *P. leo*, leopard *P. pardus*, snow leopard *P. uncia* and cheetah *Acinonyx jubatus*), while jaguar (*P. onca*) is Near Threatened and puma (*Puma concolor*) is Least Concern (IUCN, 2016). Local populations and subspecies are often worse off than the species, such as three tiger subspecies already extinct and a fourth one possibly extinct in the wild (Goodrich et al., 2015). Most leopard subspecies are classified as Endangered and Critically Endangered due to ever accelerating prey depletion, loss and fragmentation of habitats from human encroachment, and poaching (Jacobson et al., 2016; Stein et al., 2016).

Despite close attention from scientists and practitioners to resolution of conflicts between humans and big cats, some practical aspects remain poorly understood. One of them is the role of shepherds and guarding dogs (*Canis familiaris*) as the most common non-lethal method of reducing losses of grazing livestock (Dar et al., 2009; Abade, Macdonald & Dickman, 2014; Kabir et al., 2014). The presence of shepherds is believed to improve protection and alleviate depredation (Rosas-Rosas, Bender & Valdez, 2010; Fynn et al., 2016), shown when the absence of shepherds during the busy seasons of crop growing and harvesting leads to higher losses of unattended livestock from big cats (Sangay & Vernes, 2008; Kuiper et al., 2015). Furthermore, the presence of shepherds can be ineffectual if they are few, inattentive, unable to spot big cats or prevent their attacks, or if herding is done by children (Ogada et al., 2003; Woodroffe et al., 2007; Mosalagae & Mogotsi, 2013; Tumenta et al., 2013; Abade, Macdonald & Dickman, 2014; Johansson et al., 2015; Khorozyan et al., 2015b; Kuiper et al., 2015). Training of guarding dogs, which includes professionally designed and applied techniques to make dogs repel carnivores from livestock, is expensive and demanding in regard to quality dogs, time, professional resources and local acceptance (VerCauteren et al., 2012). Because of these limitations, trained guarding dogs are seldom available in developing countries, with a few exceptions like Namibia (Potgieter et al., 2013; Potgieter, Kerley & Marker, 2016). For this reason, dogs often fail to deter big cats and can be killed by them (Ogada et al., 2003; Kolowski & Holekamp, 2006; Holmern, Nyahongo & Røskaft, 2007; Woodroffe et al., 2007; Dar et al., 2009; Abade, Macdonald & Dickman, 2014; Kabir et al., 2014; Khorozyan et al., 2015b). Also, dogs are among the most preferred prey species for big cats, especially leopards, in anthropogenic prey-lean areas (Edgaonkar & Chellam, 2002; Athreya et al., 2015; Shehzad et al., 2015; Athreya et al., 2016). In general, more studies are required to assess the efficacy of shepherds and dogs and to find ways for their improvement in developing countries where most of depredation events occur (Bauer, De Iongh & Sogbohossou, 2010).

In this paper, we consider the effects of shepherds and guarding dogs on livestock losses to leopard attacks and propose appropriate conservation measures near Golestan National Park, Iran. This protected area retains an estimated 23–42 individuals, which is the largest protected population of the globally endangered Persian leopard (*P.p. ciscaucasica* = *P.p. saxicolor*) in the Middle East (Hamidi et al., 2014). The estimate of the Persian leopard population size is 800–1,200 individuals in Iran and neighboring countries (Turkmenistan, Afghanistan, the Caucasus countries, eastern Turkey and Iraq), of which ca. 70% (550–850 individuals) live in Iran (Kiabi et al., 2002; Khorozyan, 2014). The main threats to Persian leopards are prey depletion and poaching provoked by depredation (Karami, Ghadirian & Faizolahi, 2012; Khorozyan et al., 2015a; Khorozyan et al., 2015b; Ghoddousi et al., 2016; Babrgir, Farhadinia & Moqanaki, 2017). Apart from the leopard, Iran holds a number of other carnivores, which may kill livestock and get into conflict with humans: gray wolf (*Canis lupus*), jackal (*C. aureus*), brown bear (*Ursus arctos*), striped hyena (*Hyaena hyaena*), cheetah and caracal (*Caracal caracal*). Livestock depredation by wolves is much more common in Iran (Behdarvand et al., 2014), but we address only leopard attacks taking into account the top conservation status of leopard and Golestan National Park. Thus, resolution of human-leopard conflicts in Iran is essential to save this top predator, extend it to other problem species, and to improve relationships between local livelihoods and biodiversity conservation in protected areas.

## Materials and Methods

### Study area

**Figure 1:**
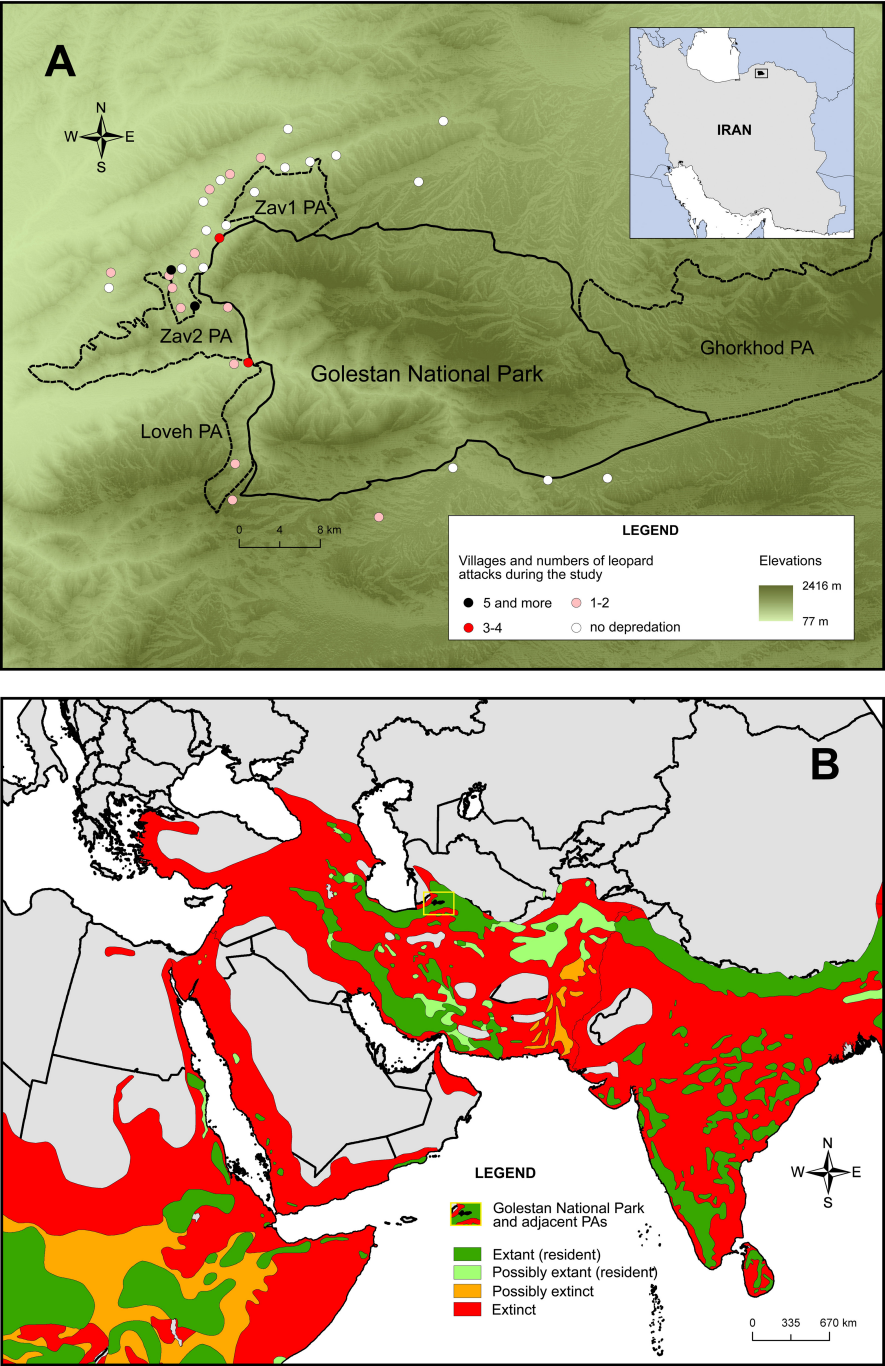
Study villages and leopard (*Panthera pardus*) attacks on sheep and goats around Golestan National Park and adjacent protected areas (PA) (A) and the current leopard distribution area (B). Source: Stein et al. (2016).

We surveyed all 34 villages around Golestan National Park (thereafter Golestan, 874.02 km^2^) and the adjoining Ghorkhod Protected Area (431.50 km^2^), Zav 1 Protected Area (50.08 km^2^), Zav 2 Protected Area (93.15 km^2^) and Loveh Protected Area (35.89 km^2^) in north-eastern Iran (Fig. 1). Golestan was established in 1957 as a reserve, which was then upgraded to the first national park of Iran in 1967 and became a UNESCO biosphere reserve in 1976. The main landscape zones are lush humid temperate Hyrcanian forest in the west, montane steppe in the central part and semi-desert in the east (Darvishsefat, 2006).

Most villages are located in the forest zone, with a few more in the steppe and semi-desert zones towards the east (Fig. 1). There are no villages inside Golestan and livestock grazing is not permitted in this park, but it illegally occurs along the park boundaries. In contrast, protected areas and wildlife refuges in Iran do allow some economic activities, e.g., livestock grazing, and may contain villages, as in the case with protected areas near Golestan (Darvishsefat, 2006).

### Herding

Local people of Turkmen, Persian, Baloch and Kurdish ethnic groups raise cattle (*Bos taurus*) and small stock (sheep *Ovis aries* and goats *Capra hircus*), and keep dogs to guard livestock. In all studied villages, grazing small stock are usually accompanied by unarmed shepherds with or without dogs, whereas cattle are often left unattended (Khorozyan et al., 2015b; Ghoddousi et al., 2016). It means that shepherds and dogs are used uniformly and not only in villages where carnivore, including leopard, attacks are possible. Traditionally, in each village small stock are grazed on a specifically designated rangeland. Thus, small stock from different flocks and villages do not compete for grazing grounds. Also, cattle and small stock graze in different areas and therefore do not interfere with each other (Ghoddousi et al., 2016).

In forests, livestock graze in the mountains in daytime and stay in village sheds at night. During summer, livestock graze near villages on wheat stubble fields and return to the mountains in early autumn when fields are trampled down or converted into rice paddies (Khorozyan et al., 2015b). In steppes, where transhumance is practiced, small stock do not return to villages for many days during summer (Ghoddousi et al., 2016). Grazing in the mountains is essential as pastures near villages are limited (median 200 ha/village; Khorozyan et al., 2015b). During winter, small stock are generally kept in sheds when snow cover is deep but graze outside villages or in areas where snow melts fast (southern or sunlit slopes) during mild winters.

### Guarding dogs

Dogs used for livestock guarding represent mixed-breed dogs of unknown parentage, with diverse body sizes, colors, physical and behavioral characteristics. With a transition from nomadic to settled lifestyle, local people let their Central Asian shepherd dogs mix with other dogs and thus diminish their original disposition for guarding. These dogs are not trained to deter leopards or other large carnivores and they show a stronger bond with people than with livestock (I Khorozyan, pers. obs., 2013). Dogs are generally human-friendly as they are raised among people, without close interactions with livestock or other domestic animals. According to local cultural norms, dogs can be only exchanged or gifted, but not sold or bought as a commodity, which prevents the purchase of quality dogs for guarding and development of dog training facilities.

### Study design and data acquisition

As we aimed to investigate the effects of shepherds and dogs, in this study we used data only on small stock. We considered sheep and goats as a single livestock species because they graze together and they are equally prone to leopard attacks (Khorozyan et al., 2015b). We recorded leopard attacks on small stock from the periods of March 2012–March 2013 and September 2014–January 2016 using structured questionnaire surveys of village heads and council members (*n* = 136) and reports from livestock owners (*n* = 29). The same researchers undertook these surveys in the same villages during these two study periods. Attack records did not overlap in households and time and we verified them by cross-checking with unrelated fellow villagers from other households. More details on the questionnaire surveys, including protocol forms, are provided in Khorozyan et al. (2015b). Local people are well skilled in discriminating leopard signs from those of the wolf, jackal, brown bear and striped hyena, which also may kill livestock in this area; therefore, we treated their information as reliable (Khorozyan et al., 2015b). Villagers were not motivated to inflate livestock losses because they did not receive compensation and, being traditionally sensitive, did not attempt to attract attention.

This project was reviewed and approved by Iranian Department of Environment (DoE), Golestan National Park and Persian Wildlife Heritage Foundation in terms of project design and communication with respondents. The written permit was issued by DoE. All people whom we asked to participate gave their verbal consent, therefore filling in a questionnaire form for a respondent signified his consent. No written consent was obtained in an attempt to establish good unofficial relationships with culturally sensitive local people, which was essential to ensure study feasibility. The respondents were informed beforehand about the purpose of questionnaire surveys, anonymity and security of their information and that this study was unrelated to governmental programs such as compensation or environmental compliance schemes. Interviews were conducted in full compliance with local traditions and ethical requirements, with local scientists Mah.S. and Mob.S. being fully involved as the Turkmen/Persian/English translators. No animal handling was conducted.

In each attack, we recorded the following variables: village, number of sheep and goats killed per attack, season, number of small stock kept in the village, ethnic groups in the village, shortest distance from the village to the nearest protected area, and presence-absence of shepherds, dogs and previous (in 2007–2011) depredation in the village. We considered two or more individuals killed per attack as surplus killing. We assigned seasons as spring (March–May), summer (June–August), autumn (September–November) and winter (December–February). Villagers did not remember the exact dates of losses, but recalled the seasons readily. We obtained the numbers of small stock from the questionnaire surveys and measured the distances from villages to the nearest protected areas in ArcGIS 10.1 (Khorozyan et al., 2015b). As small stock graze close to villages with a radius of less than 2.5 km (Khorozyan et al., 2015a; Ghoddousi et al., 2016), we assumed that the location of villages reliably indicates the location of depredation sites. As villages were not always monoethnic, we quantified ethnic groups as the presence (1) and absence (0) of Turkmens, Persians, Balochis and Kurds in villages. We categorized the presence or absence of shepherds and dogs in each attack as 1 for presence and 0 for absence. We also used the questionnaire surveys to record small stock depredation by leopards during the past five years, 2007–2011. As interviewed people did not remember the numbers of killed individuals in such old records, we quantified previous depredation as present (1) or absent (0).

### Data analysis

We used the number of sheep and goats killed per attack as a response variable and the other variables as the predictors. We applied Kruskal–Wallis and Mann–Whitney tests to compare samples, *χ*^2^ test to compare frequencies, Cook’s statistic D > 1 to identify outliers and Variance Inflation Factor (VIF) > 3 to indicate strong multicollinearity between predictors (Zuur, Ieno & Elphick, 2010; Hawkins, 2014). We used Fisher’s exact test to check the association between the presence-absence of shepherds, dogs, previous depredation and ethnic groups because the sample size was in some cases less than 5 (Johansson et al., 2015). As the number of sheep and goats killed per attack was a count statistic, we used Poisson Generalized Linear Model (GLM) to study its relationships with predictors and their interactions (O’Hara & Kotze, 2010). We checked the Poisson distribution of the number of sheep and goats killed per attack by Kolmogorov–Smirnov *z* test and by checking the equality of sample mean and variance. We ranked GLM models according to their Akaike Information Criterion corrected for small sample size (AIC_c_), which has lower values in better models. We selected the best models as those having ΔAIC_c_ < 2, in which model delta ΔAIC_c_ is the difference between a given model’s AIC_c_ and the best model’s minimum AIC_c_ (Burnham & Anderson, 2002). We also calculated the Akaike weight (w_i_) as the probability that the i-th model is the best model (Symonds & Moussalli, 2011). We used the odds ratio exp(slope) to estimate the strength of predictor effects on the number of sheep and goats killed per attack in GLM models, indicating no effect when the odds ratio is around one, negative effect when the ratio is less than one and positive effect when the ratio is higher than one (Woodroffe et al., 2007; Khorozyan et al., 2015b). We performed all statistical tests in IBM SPSS Statistics 23.0 (IBM Corp., Armonk NY, USA).

## Results

We recorded 39 attacks of leopards on small stock, which indicated a total loss of 31 sheep and 36 goats in 17 villages (Fig. 1; Table S1). These attacks included only those, on which we knew about the presence-absence of shepherds and dogs, and actual losses of sheep and goats during the study period were much higher (154 sheep and goats). All animals were killed while grazing and mostly (64 out of 67 individuals, 95.5%) around villages located in forests to the west of Golestan.

The numbers of sheep and goats killed per attack did not differ between villages (Kruskal–Wallis *χ*^2^ = 20.217, *p* = 0.211), but total numbers of sheep and goats lost per village varied widely from 1 to 18 (mean 3.9 ± 1.2) individuals. The numbers of sheep and goats killed per attack did not change seasonally (*χ*^2^ = 1.216, *p* = 0.749). The numbers of attacks were low in summer (*n* = 3) and higher in spring (*n* = 10), autumn (*n* = 12) and winter (*n* = 14), and this difference was marginally significant (*χ*^2^ = 7.051, *p* = 0.070). Total losses of sheep and goats were significantly lower in summer (*n* = 4) than in other seasons (*n* = 12–30; *χ*^2^ = 22.612, *p* < 0.001). The presence-absence of shepherds and dogs did not vary between seasons (*χ*^2^ = 3.764, *p* = 0.288 and *χ*^2^ = 3.595, *p* = 0.309, respectively). Ethnic groups in villages did not influence the numbers of attacks, losses of sheep and goats and numbers of sheep and goats killed per attack (Mann–Whitney U varied from seven to 80, p from 0.091 to 0.956). The presence-absence of shepherds and dogs were similar in all ethnic groups (Fisher’s exact test, *p* varied from 0.563 to 1.000).

**Figure 2:**
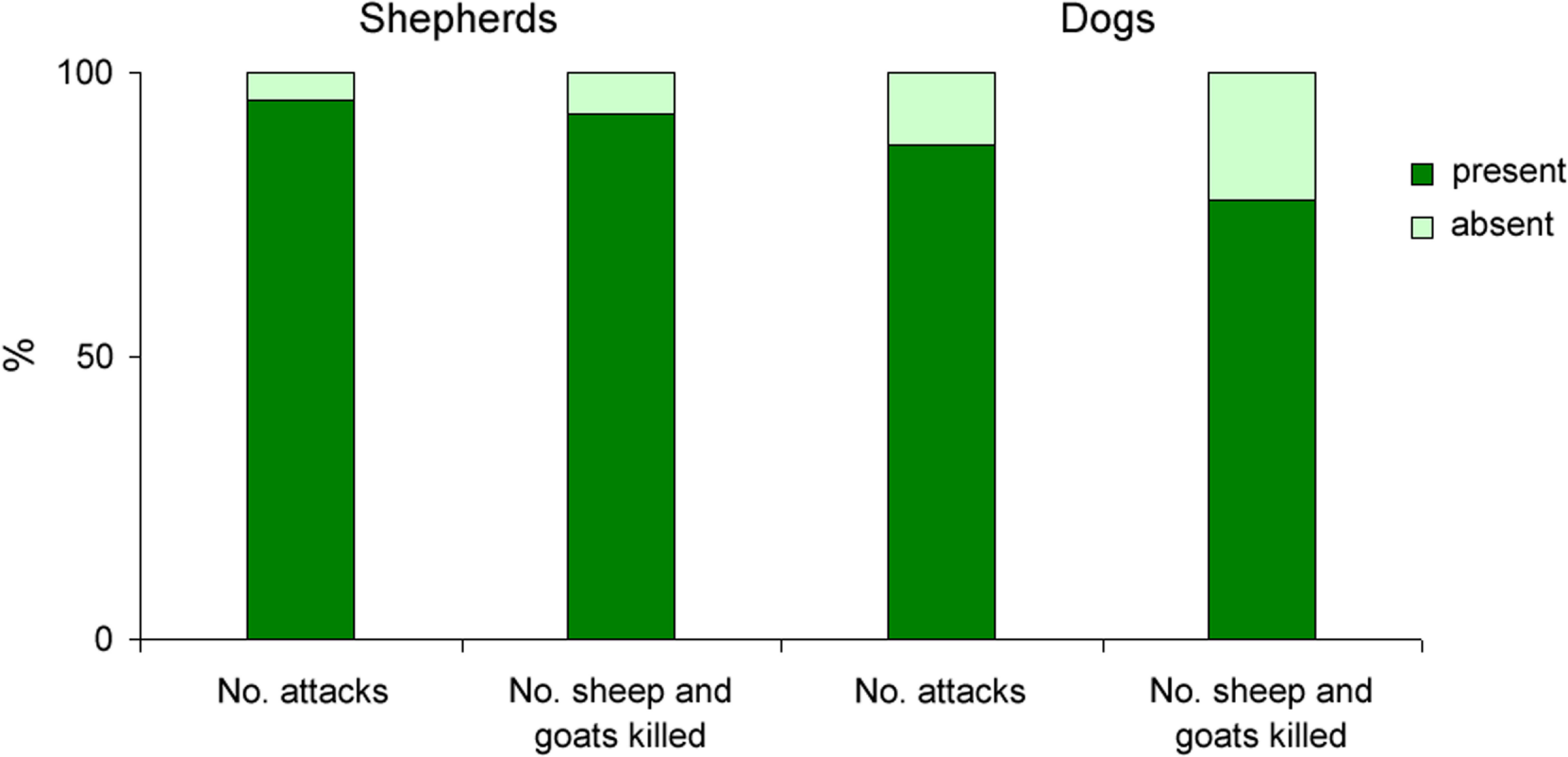
The percentages of leopard attacks on sheep and goats and numbers of sheep and goats killed during the presence and absence of shepherds and dogs.

Shepherds were present in 37 attacks (94.9%), which contained a loss of 62 out of 67 (92.5%) sheep and goats (Fig. 2). Dogs were present in 34 attacks (87.2%), which contained a loss of 52 out of 67 (77.6%) sheep and goats (Fig. 2). The numbers of sheep and goats killed per attack were not associated with the presence or absence of shepherds (Mann–Whitney *U* = 27.5, *p* = 0.457) and previous depredation in 2007–2011 (*U* = 140.5, *p* = 0.859), but were marginally higher when dogs were absent (3.0 ± 0.8 individuals/attack vs. 1.5 ± 0.2 individuals/attack when dogs present; *U* = 49, *p* = 0.063). Previous depredation in villages marginally increased the presence of shepherds (Fisher’s exact text, *p* = 0.061), but did not affect that of dogs (*p* = 1.000). Dog presence was closely related to shepherd presence (33 attacks, 84.6%), but the absence of dogs was not associated with shepherds (5 attacks with shepherds present and 1 attack with shepherds absent; Fisher’s exact test, *p* = 0.243).

Total numbers of sheep and goats lost per village were much higher with both shepherds and dogs present (*n* = 51) than with shepherds without dogs (*n* = 11), dogs without shepherds (*n* = 1) and no shepherds, no dogs (*n* = 4). This difference was highly significant (*χ*^2^ = 96.522, *p* < 0.001). The numbers of leopard attacks were also much higher with both shepherds and dogs present (*n* = 33) than with shepherds without dogs (*n* = 4), dogs without shepherds (*n* = 1) and no dogs, no shepherds (*n* = 1). This difference was also highly significant (*χ*^2^ = 74.538, *p* < 0.001). However, the numbers of sheep and goats killed per attack did not differ between these four groups of the presence-absence of shepherds and dogs (Kruskal–Wallis *χ*^2^ = 4.743, *p* = 0.192).

The number of sheep and goats killed per attack met the conditions of Poisson distribution (Kolmogorov–Smirnov *z* = 1.278, *p* = 0.076; mean = 1.72 sheep killed/attack and variance =2.16 sheep killed/attack). Out of 49 GLM models, the best model was the one, which described the dependence of the number of sheep and goats killed per attack on the interaction between dog presence-absence and distances to the nearest protected area (Table 1). According to the best model, the absence of dogs made leopards kill significantly more sheep and goats per attack (surplus killing) in villages located remotely from protected areas, but the presence of dogs did not affect the numbers of sheep and goats killed per attack in villages adjacent to protected areas. However, the number of attacks with dogs present (*n* = 34) was much higher than the number of attacks without dogs (*n* = 5), which led to higher total losses to leopard depredation with dogs (*n* = 52) than without dogs (*n* = 15).

**Table 1:** The top five GLM models applied in this study to show the effects of predictors and their interactions on the number of sheep and goats killed per attack. Abbreviations: AIC_c_, Akaike Information Criterion corrected for small sample size; ΔAIC_c_, model delta; OR, odds ratio; PA distance, distance to the nearest protected area; SE, standard error; *w*_i_, Akaike weight of the i-th model; #, model number. * The slope was set to zero as presence was used as a categorical reference for absence.

#	First predictor	Second predictor	Slope *β* ± SE	OR	AIC_c_	ΔAIC_c_	*w*_i_
1	PA distance	Dog presence	−0.06 ± 0.14	0.94	120.11	0.00	0.37
		Dog absence	3.99 ± 1.70	53.78			
2	Dog presence*		0	1	123.39	3.27	0.07
	Dog absence		0.67 ± 0.29	1.96			
3	Persian ethnic group*	Dog presence	0	1	123.63	3.52	0.06
	Non-Persian ethnic group	Dog presence	0.53 ± 0.38	1.69			
		Dog absence	1.10 ± 0.44	3.00			
4	Kurdish ethnic group*	Dog presence	0	1	123.95	3.84	0.05
	Non-Kurdish ethnic group	Dog presence	0.51 ± 0.41	1.67			
		Dog absence	1.10 ± 0.46	3.00			
5	Persian ethnic group*		0	1	124.59	4.48	0.04
	Non-Persian ethnic group		0.64 ± 0.38	1.90			

## Discussion

In our study, leopards killed most sheep and goats in forests to the west of Golestan. This is in line with our earlier studies in this area, which demonstrated that the highest levels of leopard depredation on cattle, sheep, goats and dogs are recorded in forests due to the poor health of affected domestic animals, humidity and closer distances to protected areas (Khorozyan et al., 2015a; Khorozyan et al., 2015b). Most of the annual livestock losses are incurred in villages located along the boundaries of Golestan and adjacent protected areas where the core leopard population lives (Hamidi et al., 2014; Khorozyan et al., 2015a; Khorozyan et al., 2015b; Ghoddousi et al., 2016). Leopards live throughout Golestan and not only in its western, forested part (Hamidi et al., 2014), but kill livestock grazing in forests near or inside protected areas, not in villages (Khorozyan et al., 2015b; Ghoddousi et al., 2016). Our study confirmed that the numbers of leopard attacks and small stock losses were minimal during summer when small stock grazed near villages and sharply increased in other seasons, especially autumn and winter, when small stock moved deeper into forests and often entered protected areas.

In Golestan, sheep and goats have been mostly guarded by shepherds and dogs regardless of season and ethnic group. Despite this, most of the leopard attacks and losses of sheep and goats in this study occurred when these domestic animals were accompanied by shepherds and dogs (Fig. 2). It means that local husbandry practices are ineffectual and the mere presence of shepherds and guarding dogs near small stock is not enough to secure protection. Although leopards tended to kill more sheep and goats per attack when dogs were absent (surplus killing) in villages distant from protected areas, 77.6% of losses were inflicted when dogs were present, mainly in villages located close to protected areas (Table 1; Fig. 2). We suggest that dogs may not help mitigate depredation in villages near protected areas, where pressure from leopard depredation is naturally high, but may reduce depredation in remote areas where leopard attacks are scarce.

**Figure 3:**
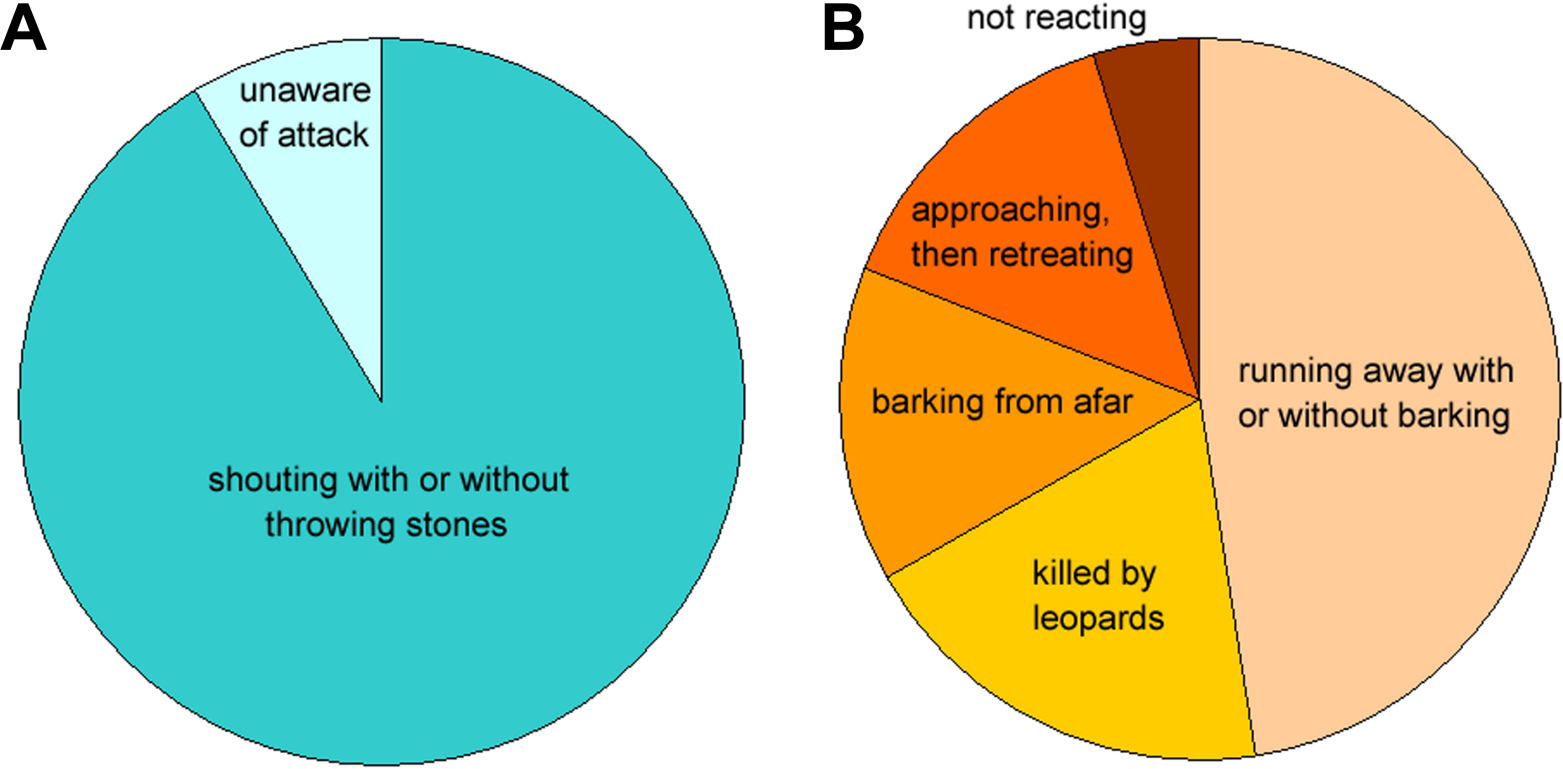
Responses of shepherds (A) and dogs (B) to leopard attacks on small stock as described in the earlier study in Golestan National Park (Khorozyan et al., 2015b).

Dogs in Golestan villages are not trained to chase carnivores, but serve to alert shepherds of danger. This is a common practice in rural communities where dog training is not practiced (Ogada et al., 2003; Abade, Macdonald & Dickman, 2014), but even in this case dogs may succeed to reduce depredation by as much as 63% (Woodroffe et al., 2007). In Golestan villages, however, local dogs fail to deter leopards by running away, barking from afar, retreating and not reacting, and sometimes are killed by leopards (Fig. 3; Khorozyan et al., 2015b). Timidity of dogs may be caused by inappropriate rearing and lack of training, which lead to strong fidelity of dogs to people and a weak bond between dogs and livestock (VerCauteren et al., 2012; Potgieter et al., 2013). Such behavior is not only ineffectual for guarding, but may even provoke leopards to pounce on dogs and small stock, especially in mountainous relief and dense forest, which favor depredation from ambush. Indeed, in five out of 39 attacks (12.8%) leopards also killed guarding dogs. Although leopards kill guarding dogs in Golestan, the dogs are rarely consumed (Ghoddousi et al., 2016; Sharbafi et al., 2016). This may indicate that predatory events are driven by defensive behaviors or instigated by the dog’s fleeing instincts triggering the predatory sequences of the leopards, rather than driven by hunger.

As dog training is not customary in Golestan, local villagers suggest reducing human-leopard conflicts through governmental actions, e.g., compensation of livestock losses, park fencing and translocation of problem individuals, but not by improving husbandry. Local villagers know that their dogs are mostly ineffectual against leopards, but keep on using them believing that livestock losses and even attacks on humans would be higher without dogs (also see Dar et al., 2009; Kabir et al., 2014). Considering the importance of keeping local traditions and cultural norms, we suggest two practical, low-cost and socially acceptable improvements in small stock husbandry in Golestan villages: (1) strengthening the dog-livestock bond; and (2) using only best available dogs for guarding. Keeping a strong social bond between a pup and livestock is an essential prerequisite to raise a good guarding dog, which is achievable mainly when pups are 3–12 months old and weaned (VerCauteren et al., 2012). It is important to keep pups present during livestock feeding (especially bottle-feeding of the young), shed cleaning and other procedures, and leave them overnight in sheds. The introduction of pups to pastures and their integration with grazing animals should begin at the age of 6–7 months and continue by gradually reducing the levels of human supervision (VerCauteren et al., 2012). These practices are often implemented in Golestan villages, yet unsuccessfully, possibly meaning that the role of human supervision still remains high and dog quality can be more important and relevant in this area than dog training. The best available guarding dogs are those, which are large, strong, healthy, steadfast, obedient to the shepherd and show no symptoms of chasing wildlife, biting livestock or attacking humans (VerCauteren et al., 2012; Potgieter et al., 2013; Potgieter, Kerley & Marker, 2016). It is also essential for good guarding dogs to stay with livestock even in the presence of carnivores and to remain aggressive towards larger carnivores (VerCauteren et al., 2012). These characteristics are generally lacking in most of available guarding dogs in Golestan. Shepherds may be tempted to increase the number of untrained dogs per flock instead of selecting the best individuals. However, due to the stealthy behavior of hunting leopards this would likely stimulate more attacks. Although dogs do not attack livestock in Golestan, they still can harass or kill small wildlife or transmit diseases (Vanak & Gompper, 2009; Hughes & Macdonald, 2013; Potgieter, Kerley & Marker, 2016). In this sense, we urge not the use of more dogs, but of better dogs, which may also improve conservation by ensuring safety and health of wildlife populations.

Shepherds in study villages are involved full-time in herding and do not leave small stock unattended during the periods of crop growing and harvesting as it is observed elsewhere (Sangay & Vernes, 2008; Kuiper et al., 2015). During the earlier study in Golestan, shepherds detected leopard attacks in most cases in spite of rough and forested landscape that might reduce visibility (Fig. 3; Khorozyan et al., 2015b). Being attentive and responsible, shepherds nonetheless failed to deter leopards because they were unarmed and could only shout and throw stones in vain. Carrying firearms is prohibited for shepherds in order to prevent retaliatory killing as the leopard is endangered and protected in Iran (Karami, Ghadirian & Faizolahi, 2012). No deterrents, e.g., fog horns and rubber bullets, are used, but their application is promising in this area. Depredation increases when livestock is attended by children (Woodroffe et al., 2007; Tumenta et al., 2013), but this is not practiced in Golestan as children go to school and may help their families only during holidays. As it is often the case in developing countries (Mosalagae & Mogotsi, 2013), shepherds in Golestan villages are usually old and often illiterate as youth are not motivated to do livestock herding and rather search for jobs in towns.

Current herding practices need to be optimized considering that the availability of shepherds is very limited in Golestan villages. To address this, we suggest to aggregate smaller flocks into larger ones and to allow available shepherds herd these larger flocks together. In other words, one shepherd with 200 stock heads, the second shepherd with 400 heads and the third shepherd with 300 heads can consolidate into a large flock of 900 heads herded by three shepherds. For example, a good solution could be the guarding of one large flock of sheep and goats by 2–3 shepherds and at least 2–3 good guarding dogs. This build-up approach is practical not only within villages, but also in villages situated near each other, especially in villages clustered to the west of Golestan where most depredation cases occur (Fig. 1). Although the effectiveness of this measure is not assessed yet, we see this approach as most practical and feasible because it does not require for additional resources and relies solely on re-grouping of available shepherds and small stock. Joint herding by several shepherds is expected to strengthen mutual psychological support, break the work monotony with communication, stimulate collective behavior, offer sharing of responsibilities over stock safety, and improve protective capacities of unarmed shepherds. Previous research on this topic is very limited, but it shows that several shepherds per flock tend to reduce depredation by African large carnivores, including leopards, in comparison with one shepherd per flock (Ogada et al., 2003; Kolowski & Holekamp, 2006; Kuiper et al., 2015). More data is needed to know whether this approach is generally applicable or whether it is biased towards carnivore species or areas.

One of the most common recommendations to improving herding practices is to avoid areas with dense vegetation, especially near water, where the depredation risk is particularly high (Rosas-Rosas, Bender & Valdez, 2010; Abade, Macdonald & Dickman, 2014). However, it is of limited value for our study area where montane humid forest provides optimal conditions for ambush hunting almost everywhere. Instead, it is reasonable to recommend avoiding areas close to Golestan and central, core parts of neighboring protected areas where leopards are more likely to attack sheep and goats (Khorozyan et al., 2015b; Ghoddousi et al., 2016).

We acknowledge some limitations of this study. The first is a rather small sample size of leopard attacks. This is a reflection of the pattern of leopard depredation on livestock in Golestan villages, which is sporadic but spread over many villages. As a result, our depredation data were limited to one to eight records per village and spread across 17 villages (Table S1). Second, it was not possible to study the effect of leopard densities and distribution of individual leopards around villages on sheep and goat losses as relevant information is lacking in Golestan. The third limitation is that this study did not have controls (shepherd vs. no shepherd, and dogs vs. no dogs) for the same flocks of sheep and goats as shepherds traditionally herd them and it is practically impossible to ensure the absence of shepherds for control. Dogs are more likely to be absent than shepherds, yet they are widely used and the paucity of “no dog” cases is an obstacle for comprehensive case-control assessments of dog effectiveness. This study nevertheless indicates that leopards are predisposed for surplus killing of sheep and goats when dogs are absent, but this aspect requires more long-term research.

The leopard is a globally recognized flagship of conservation, particularly in the Middle East where it represents the last surviving big cat (Stuart & Stuart, 2008; Karami, Ghadirian & Faizolahi, 2012). We believe that this study will make a tangible contribution to mitigation of human-leopard conflicts in Iran and other areas within the leopard’s range in Asia and Africa where livestock husbandry practices are similar and conflicts are common.

## Conclusions

In order to fill a gap in knowledge on the role of shepherds and guarding dogs in livestock depredation by carnivores, we conducted a study of leopard attacks on sheep and goats. Albeit leopards tended to kill more sheep and goats per attack when dogs were absent, they still caused most losses in total when dogs were present. These results indicate that local husbandry practices are ineffectual and the mere presence of shepherds and guarding dogs is not enough to secure protection. To improve the performance of available husbandry practices, we suggest that dogs are raised to create a strong social bond with livestock, shepherds use only best available dogs, small flocks are aggregated into larger ones and available shepherds herd these larger flocks together. Use of deterrents and avoidance of areas close to Golestan and in central, core areas of neighboring protected areas is also essential to keep losses down.

##  Supplemental Information

10.7717/peerj.3049/supp-1 Table S1 Information on 39 attacks of leopards (*Panthera pardus*) on sheep and goats in villages around Golestan National Park, Iran

## Additional Information and Declarations

### Data Availability

The following information was supplied regarding data availability: The raw data has been supplied as a Table S1.
