# Supplementary material for: Effects of shepherds and dogs on livestock depredation by leopards (Panthera pardus) in north-eastern Iran
Source: PeerJ. 2017 Feb 23;5:e3049. doi: 10.7717/peerj.3049 (PMC5326547; doi:10.7717/peerj.3049)
Supplement: Table S1 [file peerj-05-3049-s001.pdf]

Table S1. Information on 39 attacks of leopards (*Panthera pardus*) on sheep and goats in villages around Golestan National Park, Iran.

Attack #	Village	Number of sheep and goats killed per attack	Season	No. living small stock/village	Ethnic groups				Distance from village to protected areas, km	Presence (Yes) or absence (No)		
					Turkmen	Persian	Balochi	Kurdish		Shepherds	Dogs	Previous depredation in village
1	Bidak	1	autumn	6000				Yes	3.26	Yes	Yes	No
2	Dasht Shad	1	autumn	20000		Yes		Yes	0.67	Yes	Yes	Yes
3		1	winter							Yes	Yes	
4	Domanli	5	winter	1000	Yes				0.28	Yes	No	Yes
5	Gharanki Jangel	2	spring	600	Yes				0.88	Yes	Yes	No
6	Ghareh Yesar Bala	2	summer	300	Yes		Yes		2.89	Yes	Yes	No
7	Ghodeneh Bala	4	winter	400	Yes				1.81	No	No	No
8	Ghoshcheshmeh	1	spring	600	Yes				0.00	Yes	No	Yes
9	Khojeh Yapaghi	1	winter	500	Yes				1.72	Yes	Yes	No
10		1	winter							Yes	Yes	
11	Kondoskouh	1	spring	1200		Yes			0.00	Yes	Yes	Yes
12	Orjanli	7	winter	1500	Yes				1.00	Yes	Yes	Yes
13		1	autumn							Yes	Yes	
14		1	summer							Yes	Yes	
15		2	autumn							Yes	Yes	
16		2	autumn							Yes	Yes	
17		1	autumn							Yes	Yes	
18		1	winter							Yes	Yes	
19	Souvar Bala	1	spring	1000	Yes				0.00	Yes	No	Yes
20		6	autumn							Yes	Yes	
21		1	winter							Yes	Yes	
22		4	winter							Yes	No	
23		2	autumn							Yes	Yes	
24		2	autumn							Yes	Yes	
25		1	autumn							Yes	Yes	
26		1	autumn							Yes	Yes	

Attack #	Village	Number of sheep and goats killed per attack	Season	No. living small stock/village	Ethnic groups				Distance from village to protected areas, km	Presence (Yes) or absence (No)		
					Turkmen	Persian	Balochi	Kurdish		Shepherds	Dogs	Previous depredation in village
27	Souvar Paiin	1	spring	700	Yes				0.19	Yes	Yes	No
28		1	summer							Yes	Yes	
29	Souvar Vassat	2	spring	400	Yes				0.78	Yes	Yes	Yes
30	Tangerah	1	spring	450	Yes	Yes	Yes	Yes	0.00	Yes	Yes	Yes
31		1	winter							Yes	Yes	
32		1	winter							Yes	Yes	
33		1	spring							Yes	Yes	
34	Terjenli	1	winter	500	Yes	Yes	Yes		0.63	Yes	Yes	Yes
35	Yekeh Ghozz Bala	1	winter	3700	Yes				3.37	No	Yes	No
36		1	winter							Yes	Yes	
37	Zav-e-Paiin	1	spring	120	Yes				0.20	Yes	Yes	Yes
38		1	spring							Yes	Yes	
39		1	autumn							Yes	Yes	
